# Success story of GLP-1 agonist (Liraglutide) treatment in someone with type 1 diabetes: a life transformed

**DOI:** 10.1097/XCE.0000000000000293

**Published:** 2023-09-28

**Authors:** Adrian H. Heald, John Warner-Levy, Lleyton Belston, Hellena Habete-Asres, Linda Horne, Ann Metters, Martin Whyte, Martin Gibson

**Affiliations:** a Department of Diabetes and Endocrinology, Salford Royal Hospital, Salford; b The School of Medicine and Manchester Academic Health Sciences Centre, University of Manchester, Manchester; c Kings College London University, London; d Vernova, Macclesfield; e Department of Clinical and Experimental Medicine, University of Surrey, Guildford, UK

## Background

Glucagon-like peptide-1 (GLP-1) agonists are now widely used for the treatment of type 2 diabetes (T2D) [1]. To date, several reports have described improvements in glucose control with the addition of a GLP-1 agonist to the insulin regime in the treatment of type 1 diabetes (T1D) [2]. Although this remains an off-licence use of the medication, these reports do indicate potential benefits in relation to this glucose control.

GLP-1 belongs to the group of incretin peptides, and it stimulates insulin and inhibits glucagon secretion. Actions of GLP-1 also include delaying gastric emptying, reduction of appetite and induction of satiety. Furthermore, evidence mainly collected from animal models has indicated the role of GLP-1 in increasing beta cell proliferation and differentiation and in decreasing the rate of beta cell apoptosis [3].

From a physiological perspective, there is credence to the notion that a GLP-1 agonist will lower glucose levels in people with T1D as they do in T2D. GLP-1 is an endogenous hormone that regulates the secretion of both insulin and glucagon in response to meals, but GLP-1 agonists also slow gastric emptying through effects on the autonomic nervous system and act centrally to increase satiety [4]. Although it has been reported that, in the short term, Liraglutide (a GLP-1 agonist) reduces average blood glucose when added to continuous subcutaneous insulin therapy [5], evidence for the use of GLP-1 agonists in T1D is limited and varies based on the agent [6].

Liraglutide is the most widely studied GLP-1 agonist in adults with T1D, as an add-on therapy to insulin [2]. Despite the limited number of patients and varying study durations in the clinical trials, results are relatively consistent across studies [2], with an average 5 mmol/mol (0.5%) glycated haemoglobin (HbA1c) lowering when liraglutide was added on to insulin therapy. However, this is not a unanimous finding, with one study reporting that the addition of Liraglutide to normal-weight patients with T1D inadequately controlled on insulin produces no effect on HbA1C [7]. Reports that the addition of Liraglutide therapy can induce weight loss exist, likely due to the subjective increase in satiety reported by many patients, with a subsequent reduction in total calorie intake [8].

In support of the role of the GLP-1 agonist in this context, Exenatide, the first of the GLP-1 agonists to be licensed, has safety and efficacy data for up to 18 months in T1D, the longest of any GLP-1 agonist [2]. It has been reported that the addition of Exenatide to insulin therapy leads to a lowered total daily dose of insulin of up to 24 units/day [9].

Although the use of GLP-1 agonists is showing promise for lowering HbA1C in patients with T1D inadequately controlled by insulin, caution must be taken. Increased rates of symptomatic hypoglycaemia and hyperglycaemia with ketosis have been reported, which may provide issues with the use of Liraglutide in addition to insulin in clinical practice [10,11]. As a result, careful insulin titration with close supervision is recommended in patients receiving combined therapy with insulin and Liraglutide [11].

Here, we report how the addition of Liraglutide to a basal-bolus regime resulted in a positive change in glucose profile as evidenced by FreeStyle Libre glucose monitoring. We have received permission from our patient regarding publication.

## Report

First diagnosed with T1D following hospital admission with diabetic ketoacidosis, our patient has experienced challenges with their management and medication concordance in recent years despite being maintained on a basal-bolus insulin regime, with HbA1c recordings consistently above 75 mmol/mol. Monitored every 6 months, his albumin/creatinine ratio has been elevated at 8.7 mg/mmol. He works long hours in his own business and recently developed a limb-threatening foot ulcer, which has now healed.

With the addition of Liraglutide to the basal-bolus regime, by 8 weeks there was a frameshift in glucose profile as evidenced by FreeStyle glucose monitoring. This was not associated with any change in blood pressure readings.

**Fig. 1:**
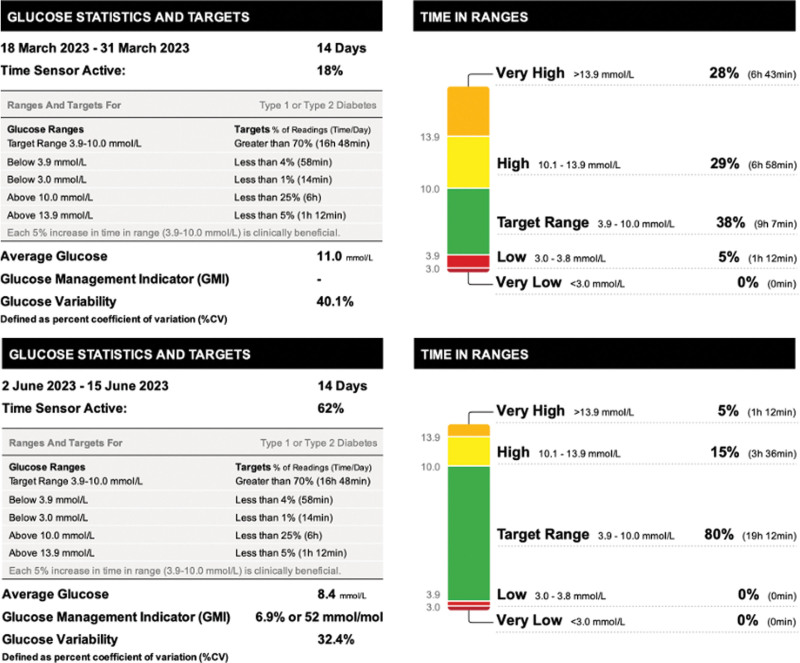
(a and b) Provide a visual comparison of glucose monitoring data between the two time periods for our patient, allowing for a greater appreciation of the improvement in time spent within the target glucose range in the second monitoring period (Liraglutide and insulin combination therapy).

**Fig. 2:**
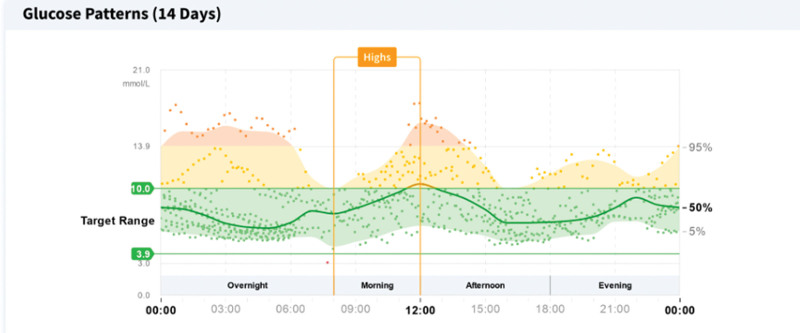
Plots glucose monitoring data for the second monitoring period on a scatter graph, providing a moving average of blood glucose levels, whilst also demonstrating the overwhelming majority of time spent by our patient with a blood glucose level within the target range.

The time spent in the range of glucose (3.9–10.0 mmol/L) increased from 38 to 80% with the percentage in the range >10.0 mmol/L decreasing from 57 to 20%. Also, the variability decreased from 40.1 to 32.4%% and estimated HbA1c from 69.9 mmol/mol to 51.9 mmol/mol (Figs. 1 and 2).

The change in glucose levels was associated with a self-reported enhancement of the sense of control of the T1D day to day. In his words, ‘I feel in control of my diabetes for the first time in 20 years – this has transformed my life. I am back to my old self again’.

We accept that a limitation of our report is that we did not include any measures of glucose handling at a tissue level or insulin sensitivity, as the initiation was part of usual care. However, we hope that our case study can inform more research in this clinically relevant area.

In conclusion, it is well documented that risk factor optimization in people with T1D is integral to improved length of life and reduced complication rate [12]. We here report a ‘good news story’ of the benefits of GLP-1 agonist treatment in T1D in relation both to quantitative glucose profile and the individual’s sense of agency in their self-management going forward.

We hope that this will assist clinicians as they make decisions in relation to complex T1D management.

## Acknowledgements

### Conflicts of interest

There are no conflicts of interest.
